# The Reformed Public House

**Published:** 1923-11

**Authors:** 


### The Reformed Public House,

SIR,—Mr. Dering is not, I believe, a Prohibitionist. He does not wish to abolish the drinking of alcoholic beverages in moderation. It follows, therefore, that in his opinion the evil is drunkenness. With this I agree, but any scheme of licensing reform should therefore be judged by its effect on drunkenness. Has the Carlisle scheme of State ownership and control, which Mr. Dering advocates, resulted in a greater decrease in drunkenness than is seen in places where licensed premises are still privately owned? The evidence does not support such a contention. Increased sobriety is, one is glad to know, practically universal, but statistics indicate that Carlisle shows no greater increase than the general average, and in fact not so great a decrease in drunkenness as many other places show.

PRIVATE ENTERPRISE.

